# A systematic review of hospital experiences of people with intellectual disability

**DOI:** 10.1186/s12913-014-0505-5

**Published:** 2014-10-25

**Authors:** Teresa Iacono, Christine Bigby, Carolyn Unsworth, Jacinta Douglas, Petya Fitzpatrick

**Affiliations:** La Trobe Rural Health School, La Trobe University, Bendigo, PO Box 199, Bendigo, Victoria 3552 Australia; Department of Social Work and Social Policy, La Trobe University, Melbourne, Australia; Department of Occupational Therapy, La Trobe University, Melbourne, Australia; Department of Human Communication Sciences, La Trobe University, Melbourne, Australia

## Abstract

**Background:**

People with intellectual disability are at risk of poor hospital experiences and outcomes. The aims were to conduct a content and quality review of research into the acute hospital experiences of both people with intellectual disabilities and their carers, and to identify research gaps.

**Method:**

A systematic search was conducted of primary research between 2009 and 2013 that addressed the experiences of the target group in general acute care hospitals. Quality appraisal tools yielded scores for quantitative and qualitative studies, and overarching themes across studies were sought.

**Results:**

Sixteen studies met inclusion criteria. Quality scores were 6/8 for a survey, and 2/11-9/11 (mean =5.25) for qualitative studies/components. Content analysis revealed seven over-arching themes covering individuals’ fear of hospital encounters, carer responsibilities, and problems with delivery of care in hospitals including staff knowledge, skills and attitudes.

**Conclusions:**

Our review of eligible papers revealed that despite 20 years of research and government initiatives, people with intellectual disability continue to have poor hospital experiences. The need for research to identify and investigate care at specific points of encounter across a hospital journey (such as admission, diagnostic testing, placement on a ward, and discharge) as well as to include people with a diversity of disabilities is discussed in terms of potential to influence policy and practice across health and disability sectors.

**Electronic supplementary material:**

The online version of this article (doi:10.1186/s12913-014-0505-5) contains supplementary material, which is available to authorized users.

## Background

Internationally, hospital and primary health care systems have come under increasing strain as a result of ageing populations and a rise in chronic disease [1]. Australia sits around the middle of OECD countries in terms of the number of hospital episodes per 1,000 population [2]. Initiatives to reduce primary health and hospital inefficiencies have led to the identification of large health inequalities, in particular for Aboriginal and Torres Strait Islanders, people with mental illness and those living in remote areas of Australia [2]. Missing from these health care initiatives are people with intellectual disability - another group who similarly experience large health and health care inequities [3]. Intellectual disability has its onset before the age of 18 years, is characterised by lifelong limitations in IQ (70 or less) and adaptive functioning and accounts for 1% to 3% of the world’s population [3]. In developed countries, people with intellectual disability (a) have higher mortality rates and shorter life expectancy than the general population, (b) have greater health care needs, (c) engage in fewer preventative health screenings, and (d) have conditions that often go undiagnosed or are mismanaged [4-7]. They are also high users of hospital services. Recent international data demonstrate that people with intellectual disability use hospital services, particularly emergency departments (ED), more often than other people, especially for conditions more appropriately treated elsewhere (such as out-patient or primary care clinics), and their stays are longer [8-10].

In Australia, there have been few attempts to compare the experiences and outcomes of hospitalisations among people with intellectual disability to those of the general population. There has, however, been some acknowledgement of their experience of health inequalities in the National Disability Strategy that is driving large scale reform of the interface between mainstream and specialist disability services and support, and has resulted in a National Disability Insurance Scheme to provide a sustainable individualised funding model to deliver disability services [11]. This strategy was the impetus for a recent state government commissioned report, which provides the most detailed analysis of hospital usage for people with intellectual disability in Australia [12]. Linking disability services and health department data, hospital separations^a^ were determined for disability clients, predominantly intellectual disability, physical disability, and acquired brain injury, each year from 2005–2010. This analysis revealed their over-representation in the hospital data, with some service users having as many as 8.7 times that of the general population. The last year of data indicated they accounted for 11% to 17% of high end hospital separations and ED presentations, yet accounted for around 68% of all bed days, 44% to 67% of separations, and around 65% of hospital costs [12].

It would seem, then, that people with intellectual disability are high frequency and costly users of hospital services. Unfortunately, there is mounting evidence that they are at particular safety risk in acute hospital settings [13] and have poor experiences, in some cases pointing to institutional discrimination [7,14,15]. Research into the hospital experiences of people with intellectual disability has been driven in the UK by a peak body report describing the disturbingly poor hospital treatment of six adults who died in hospitals in England and Wales, arguably as a result of neglect, discrimination, and mismanagement [16]. Despite government effort [17] to adopt 10 recommendations from an independent inquiry [18], there has been recent evidence of continued neglect and discrimination, with dire outcomes for hospital patients with intellectual disability [15]. Most recently, a UK population-based confidential inquiry revealed that avoidable deaths (preventable or amenable through good quality care) were more common among people with intellectual disability than the general population: 37% vs. 13% of avoidable deaths [19]. The contributors were failures in health care provision that resulted in delays in diagnosis and treatment.

The UK case studies [15] and population study reflect experiences in other countries. Iacono and Davis [14], for example, provided reports by family and paid carers of people with intellectual and/or physical disabilities in Australia, which pointed to negative attitudes by hospital staff, delays in diagnostic evaluations and treatment, and reliance on family and paid carers for both advocacy and care during hospital encounters. Others have similarly explored the experience of hospital encounters for people with intellectual disability, including in Australia [20], England ([21], Scotland [22], Canada [23], and Northern Ireland [24]. Furthermore, a submission to inform a New South Wales (NSW) state health plan by a peak body indicated that the poor experiences and outcomes documented for people with intellectual disability are similarly experienced by those with other forms of disability [25]. Mounting evidence in both the grey and published literature suggests continued and serious problems during hospitalisations for people with intellectual and other types of disability and those who support them.

This review was conducted in Australia where it is particularly timely to consider the research evidence about how hospitals may meet the needs of people with disabilities. Recently, the government called for reasonable adjustments by mainstream services, including health care, to ensure equality for people with disability [11]. There is also a need to understand the nature and degree of adjustment required of hospital staff, and how these adjustments may contribute to hospital quality and safety performance [26]. In Australia, policy directives to address the needs of people with disability in hospitals have been evident only in one state [27]. These directives include reduced reliance on family and paid disability staff, and adjustments to pre- admission and discharge plans. Unfortunately, the NSW policy directive fails to compel services to make changes [25]. Similarly, other policy and practice documents have failed to address the needs of people with disability, more broadly, during hospital encounters [26].

A number of reviews have explored underlying contributors to poor hospital experiences by people with intellectual disability [13,28-30]. These reviews have similarly revealed that some people with intellectual disability are fearful of hospital encounters, there is reliance on carers during the entirety of stays, and problem attitudes and limited knowledge of hospital staff, sometimes with dire outcomes. Recommendations to ameliorate problems identified in studies reviewed by Backer, Chapman and Mitchell [13] reflect those from the UK inquiry [18]: that is, developing liaison models, especially through a disability liaison nurse, supporting carers, and improving communication. Mencap's [15] concern of little progress since these recommendations were published has been borne out in the literature, with little evidence, for example, of adopting the frequently recommended Learning Disability Liaison Nurse strategy [29].

Backer et al. [13] argued for further research into hospital access and experiences for people with intellectual disability, but there is little to indicate the value of or specific need for continued research. Previous reviews have focused on summarising findings of largely qualitative research, with little critique of their quality. Phillips [29], for example, noted the use of a critical appraisal tool, but reported methodological limitations of studies reviewed rather than providing quality scores. Bradbury-Jones et al. [30] used a dichotomised rating of weak or strong, but provided little in the way of rating criteria or reliability. Also, there has been no attempt to determine the extent to which studies have contributed specific and new information about the nature and points of hospital encounters that may present particular challenges in meeting the needs of patients with disability. Hospital encounters, or points of encounter refer to specific points on the patient’s journey through a hospitalisation, such as admission, diagnostic testing, placement on a ward, and discharge. Recommendations for adequate assessment of needs and discharge planning [13] fail to take into account the many points of encounter with staff filling varied roles, or of patient requirements as they engage in the journey from presentation to and exit from hospitals.

Prior to undertaking further research into hospital experiences, in general, a critical quality review and analysis of the points of hospital encounters are needed. Such a review promises insights into barriers and enablers of reasonable adjustments by hospital services across the patient journey. It would also provide direction for further research to ensure quality hospital care for people with intellectual disability and others made vulnerable by reliance on others for care and advocacy.

### Aims

The review addressed the issue of how the hospital system responds to adults with intellectual disability, their families and carers as reported in current research. Specific aims were to (a) understand the experiences of adults with intellectual disability using hospital systems and the views of their families and paid carers; (b) understand the experiences of hospital staff who provide care for people with intellectual disability in hospital; and (c) evaluate the quality of the research and identify gaps in the literature, in particular in terms of the types of interactions and points of encounter along the patient journey.

## Methods

Searches were conducted within Medline, CINAHL, EMBASE, Sociological Abstracts and PsycINFO, with the limiters English-language, primary peer-reviewed research articles, published between 1990 and 2013, and research with original data. Search terms are presented in Table 1. Inclusion criteria were that most of the research population had an intellectual disability (arbitrarily set at 70%), and the main topic related directly to one or more of the specific aims. Retrieved abstracts were scanned manually and potentially suitable papers further assessed against the inclusion criteria. Potential papers were assessed by three authors (TI, CU& PF) and a fourth (CB) was consulted if consensus could not be reached. Corresponding authors were contacted for papers with insufficient details to enable a decision about inclusion: these inquiries were usually about the participant groups. Reference and forward citation searches were performed on included articles and relevant literature reviews.

**Table 1:** **Search terms**

***Population***	***Hospital setting***	***Experiences***
Cognitive disability	acute care	communication difficulties
Cognitive impairment	admission	difficulties communicating
Communication disability	casualty	experience of care
Communication disorder	discharge	experience of hospital
Communication impairment	emergency care	experience of patient
Complex communication needs	emergency department	health care/healthcare
Developmental disability	hospital	experience
Intellectual disability	hospital care	health care/healthcare
Intellectual handicap	hospital staff	disparities
Intellectual impairment	inpatients	patient experience
Learning difficulty	learning disability nurse	patient-staff communication
Learning disability	medical staff	quality of care
Mental handicap	nurse	quality of health care/ healthcare
Mental retardation	nursing staff	staff-patient communication
Non-speaking	patient	treatment outcome
	outpatient	

Tools to evaluate the studies were developed from Downs and Black [31], with additions from the McMaster guidelines for the qualitative studies [32], to provide overall quality scores from a possible score of 11^b^. The first author completed quality appraisals with two other authors appraising two each for reliability (4/17 studies). Item-item agreement was 80%-100% (mean =90%); overall scores differed by one point for one of the four studies with agreement for the other three.

The data extracted were participant characteristics, including demographic details, points of hospital encounter and staff-patient interactions; these are presented in Additional file 1. Results of audits of Learning Disability Liaison Nurse (LDLN) services from two mixed methods studies [22,33] have not been reported in Additional file 1; rather only details of the qualitative components. The qualitative components/ studies were then analysed thematically for patterns of findings and gaps in the research identified. This process involved listing themes reported in each study and conducting a content analysis for grouping according to over-arching and sub-themes (completed initially by TI and independently reviewed and confirmed by CB).

## Results

A total of 614 abstracts were retrieved from the database searches and a further 94 articles identified through citation, reference, hand and general web searches. Of these, 608 articles were excluded and the remaining 100 were obtained and assessed for eligibility. The search identified 16 studies that met the inclusion criteria.

### Quantitative study

#### Characteristics and quality

The quality score for the survey component of the study by Iacono and Davis [14] was 6/8 (see Table 1). The main area of weakness was in relation to external validity in that sample representativeness was not demonstrated.

### Findings

Key findings from the survey were that, of 119 adults who had recent hospital experiences and did not have their needs met, only 12% received their correct medications and 22% did not receive them on time; 18% did not receive enough to drink; 39% could not get to the toilet when needed; and 11% could not move from their bed when needed. Most adults about whom responses were provided needed someone with them most of the day, from early till late (*n* =79), which tended to be a family or paid carer in the absence of alternatives, which was found to be associated with having their basic needs met. For 89 adults, information was provided about what to do when they left hospital; 71 had letters for their local doctor or other professional.

### Qualitative studies

#### Characteristics

Inspection of Additional file 1 reveals all studies were qualitative or had a qualitative component. Participant groups within these qualitative studies included adults with intellectual disability, and their family and/or paid carers, hospital staff, and primary care professionals. It was difficult to determine total numbers within each category given variability in details provided: Cumella and Martin [34], for example, noted that 40 participants included people with intellectual disability and other groups involved in the hospital experience, but did not indicate the sample size for each group. Points of hospital encounters varied, but many were unspecified or required extrapolation from the direct participant quotes. Frequent points of encounter were ED or acute care, and various wards, with preadmission, out-patient and X-ray departments also included; but there was no attempt to compare across these settings. Little information was provided about the interactions investigated, although when noted, they were mostly with nurses and doctors.

The appraisal scores varied from 2 to 9 (mean =5.25) out of a possible 11. Contributors to poor quality according to the appraisal tool included inadequate information on how participants were selected and failure to sample until redundancy in data was reached [35]; lack of clear evidence that data analysis was inductive [36,37]; limited reporting on the decision trail in terms of rules and how decisions were made [38]; and most often, a lack of evidence for any or more than one component of trustworthiness [39].

### Themes

Themes identified in each qualitative study/component of the 16 studies were grouped according to seven over-arching themes, presented in Table 2. These themes related largely to failures of hospitals and staff to meet patient needs. These failures appeared to arise from limited knowledge and skills of staff, as well as negative attitudes, and hospital systems failing to make required adjustments, resulting in reliance on carers for both care and advocacy for appropriate treatment. These difficulties may have contributed to the fear of hospitals experienced by people with intellectual disability. Factors that facilitated appropriate care noted in some studies, largely by adjusting to needs, appeared to be exceptions rather than common experiences.

**Table 2:** **Themes derived from the 16 qualitative studies meeting inclusion criteria**

**Overarching theme**	**Sub-themes**
Fear of the hospital encounter by people with intellectual disability	Fearful because of not knowing what to expect
	Not understanding the situation; fear of an unfamiliar situation and environment
	General fear of nurses, doctors and medical procedures, which were associated with hospitals
Failure of hospital staff to provide care	Delayed or no appropriate diagnostic procedures
	Diagnostic overshadowing (attributing symptoms to the intellectual disability)
	Failure to treat pain (and inability to identify pain in patient with intellectual disability)
	Failure to heed or respond to carer information
	Lack of discharge planning or strategies (also lack of continuity of care)
Hospital staff knowledge and skills	Lack of information about patients in terms of presenting underlying conditions
	Not knowing that people with intellectual disabilities can experience the same range of problems as others, and/or are at high risk for some conditions (e.g., epilepsy)
	Inability to deal with challenging behaviours
	Overall, lack of training in relation to intellectual disability
Poor or negative attitudes by hospital staff towards people with intellectual disability	Discrimination in denying diagnostic procedures or treatments
	Indifference to patients or their carers
	Lack of caring and poor or no communication with the person with intellectual disability and/or family or support person
Staff or system failure to adjust to the needs of people with intellectual disability	Long wait times in waiting rooms
	Inability to adjust communication to meet the person’s needs
	Failure to provide required assistance to enable a person to eat a meal or go to the toilet
	Failure to take account of differences in medication regimes across home and hospital settings, with potential serious outcomes
Carer responsibility	Over-reliance by hospital staff on family and paid carers to provide attendant care (toileting, meals), and assist with medical care (e.g., changing bandages)
	Advocating or insisting on appropriate investigations or treatment
Enhancers to appropriate hospital care	Repeat experiences of hospital staff with the same patient resulting in understanding and adjusting to their needs
	Presence of a hospital liaison person, such as LDLN
	Hospital policies and systems that address the needs of people with intellectual disabilities in the form of adjustments to systems and processes
	Willingness of hospital staff to “go beyond the call of duty” to ensure communication and meeting the person with intellectual disability’s needs

## Discussion

This review extends that of Backer et al. [13], whose focus included, but was not limited to, hospital experiences of people with intellectual disability. In addition to their 7 studies that met our selection criteria, an additional 10 were identified, 8 published since their search limit of March 2008. The search strategy used for the current study also yielded 8 more studies that met inclusion criteria than included by Bradbury-Jones et al., perhaps reflecting the use of five versus two databases and less restrictive search terms, despite both reviews having a focus on acute hospital care. In terms of quality, in the current review, the highest scores (7–9) were assigned to recent studies only [20,40], suggesting an overall improvement over time.

Themes that emerged from the studies indicate little additional insight into the nature of the problems experienced by people with intellectual disability to those in the previous reviews [30]. They continue to experience fear in relation to hospital encounters. Further, they continue to rely on paid or family carers to provide constant care so that basic needs are met, and for advocacy to ensure they receive appropriate assessments and treatments. Negative attitudes from nurses, as well as other hospital staff continue to be evident, although there remains limited information on interactions with varied staff. While researchers have repeatedly made recommendations for training of staff [14,38], in one of the most recent studies reviewed, hospital staff themselves identified their lack of knowledge and need for education about intellectual disability [33]. Worryingly, results by Ali, Scior, Ratti, Strydom, King and Hassiotis [41] suggest that people with intellectual disability from certain cultural backgrounds may be particularly at risk of discrimination. Their participants with intellectual disability suggested that their experience of lack of care was no different to that of other patients, a potential indictment on the hospital system in general.

There was also evidence supporting Mencap's [15] concern that published recommendations to support hospitals in caring for people with intellectual disabilities have not been implemented. Phillips [29], for example, located only one study evaluating LDLN services. These nurses provide specialist knowledge in intellectual disability within general hospital settings. Evaluations suggest the valuable role they play in promoting and supporting effective co-ordination of care from admission through to discharge, and in assisting hospital staff to make reasonable adjustments to meet patient needs [22,33]. The implementation of this or a similar service no doubt demonstrates a hospital’s commitment to care, also demonstrated through relevant policies and procedures, suggested by Webber, Bowers and Bigby [20] to contribute to quality care. Sustained initiatives, such as LDLN services, and policies that lead to effective strategies may rely on understanding hospital systems and resourcing. There is limited evidence of capacity to provide the types of accommodations sought in policy [15,25], and these factors appear to be missing from both health and disability service systems.

### Directions for future research

The current and previous reviews indicate an increasing research base concerning the hospital experiences of people with intellectual disability. In particular, the relatively large qualitative study by Webber et al. [20] supports findings from smaller previous studies, including those of poorer quality. There remains a need for research to address specific gaps, including comparing the experiences of people with intellectual disability with others sharing key characteristics. People with traumatic brain injury (TBI), for example, experience difficulties with executive function, self-direction and communication of various types and severity levels, resulting in their reliance on carers to navigate and support them during interactions with health care systems. Research into the experience of young people with TBI placed in residential aged care settings, post-acute care and rehabilitation, shows that this group has similar hospital care needs as people with intellectual disability [42]. Research that identifies both common and specific needs by comparing patient groups, such as intellectual disability and TBI, offers the potential to improve the patient experience for all vulnerable groups who present challenges to staff’s usual modes of operating.

Another gap identified involves the need for more research to investigate recommendations arising from studies with diverse patient groups. It is possible that such recommendations may more readily be implemented into hospital practices as they are likely to be perceived as having broader benefits. Relatedly, research that includes an exploration of hospital systems in a search for barriers and enhancers to quality health care for people with disability may reveal strategies aligned with their own quality care processes. For example, addressing medication safety for people with disability, found to be problematic in this review [14,36], arguably would have a greater chance of adoption if aligned with existing hospital safety processes. In particular, ensuring continuity of medication between a person’s hospital stay and return to residence aligns with a requirement in Australia to reconcile all medications when a patient is transferred out of hospital [43]. People with intellectual disability require an extension of this practice at the point of admission to ensure all medications are presented at admission and appropriately administered during the hospital encounter. This strategy would eliminate safety risks arising from changes in dosages or delays in medication administration during a hospital stay [14,36]. Such an adjustment is relevant to any patient on medication prior to hospitalisation, but has particular importance for groups known for polypharmacy, such the aged and those with chronic health conditions.

Further, effective and specific accommodations may be best identified by exploring issues faced by vulnerable groups at each point along the hospital journey. Hannon [44], for example, noted the benefits of a pre-admission assessment for people with intellectual disability, their carers and hospital staff. The benefits were explored in relation to the actual admission process only; further research into those accruing as the person journeys to various departments and then to discharge is needed.

Including various points of encounter would also provide the opportunity to investigate specific needs of and demands on patients, carers and various hospital staff. Studies in this review provided some insight into experiences with ED [14], including for psychiatric emergencies [40], and an X-Ray department [39], which similarly demonstrated themes presented in Table 2. Hart [35] interviewed participants about their experiences as inpatients, outpatients and day surgery, but did not compare across them. Investigation of differences across points of encounter may enable recommendations for adjustments to be made more reasonable from the perspective of hospital staff: that is, by considering differences in the nature of interactions and requirements across steps in the patient journey.

### Policy imperatives

Consideration of what constitutes reasonable adjustments have been explored within the disability literature [13,18,25], but evidence of consultation with mainstream services is lacking. The readiness with which mainstream services, such as health, accommodate to meet the needs of people with disabilities has been argued as core to the success of Australia’s National Disability Insurance Scheme [45]. Further, the Australian Disability Strategy provides a policy framework for coherence and co-ordination of mainstream and disability services to improve the capacity of mainstream services to deliver quality outcomes for people with disability, make disability issues visible to ensure their concerns are addressed in policy, and lead initiatives for greater inclusive practices [11]. Hence, there is a cross-government policy driver for Australian hospitals to evaluate their quality of care for people with disabilities. Although the international research reviewed paints a relatively gloomy picture, there is evidence of positive practices once hospital staff are provided the opportunity to learn the specific needs of patients with intellectual disability through repeat visits [14,41], or through more formal strategies, such as reliance on LDLNs in the UK [22,33].

Offering complementary specialist disability consultation or services within hospitals, such as the LDLN, reflects a hospital policy commitment to improving care for people with intellectual disability, but it is unclear if these will continue or extend beyond the few examples evident in the literature [29]. Certainly in the latest Mencap report [15], it was argued that the UK Government had lost the impetus to monitor implementation of recommendations of the independent inquiry into health care access by people with disability [18]. Sustaining such efforts would seem to require a whole-of-government and whole-of-society acceptance of the responsibility for true inclusion, especially in access to quality hospital care.

## Conclusions

Over the previous two decades, evidence of poor quality hospital care for people with intellectual disability has been mounting. Most of this evidence is qualitative in nature and of variable quality. Despite problems with the evidence base, and arguments for more research into hospital experiences and access to health care by people with intellectual disability, we have argued for a more expanded research focus. There is a need to explore specific points of encounter along the patient journey, and also extend understanding of the patient experience to other vulnerable groups, such as people with TBI. Such research, conducted in conjunction with exploration of hospital systems and processes, offers the potential to ensure quality of care for people with diverse needs. A further potential outcome of this expanded focus is to provide direct resource benefits to hospitals by identifying strategies that can be adopted readily, and reduce unnecessary and repeat presentations, and associated costs.

## Endnotes

^a^Hospital episodes that end with discharge, transfer to another service, or death.

^b^Copies of the appraisal tools are included in Additional file 2.

## Additional files

Additional file 1:
**Summary of studies meeting inclusion criteria.**
Additional file 2:
**Appraisal Checklists.**

## Abbreviations

ED: Emergency Department
LDLN: Learning disability liaison nurse
NSW: New South Wales
OECD: Organisation for Economic Co-operation and Development
TBI: Traumatic brain injury
UK: United Kingdom

## References

[1] National Health and Hospitals Reform Commission (2009). A Healthier Future for all Australians – Interim Report December 2008.

[2] Australian Institute of Health and Welfare (2007). Australian hospital statistics 2005–06. Health Services Series no 30.

[3] Balogh R, Ouellette-Kuntz H, Bourne L, Lunsky Y, Colantonio A: **Organizing healthcare services for persons with an intellectual disability (Review).** *Cochrane Database Syst Rev* 2008, **8**(4):doi:10.1002/14651858.CD007492. 10.1002/14651858.CD007492.pub2 PMC8720486 27065018

[4] Scheepers M, Kerr M, O'Hara D, Bainbridge D, Cooper SA, Davis R, Fujiura G, Heller T, Holland A, Krahn G, Lennox N, Meaney J, Weymeyer M (2005). Reducing health disparity in people with intellectual disabilities: a report from health issues special interest research group of the international association for the scientific study of intellectual disabilities. J Policy Pract Intellect Disabil.

[5] Sutherland G, Couch MA, Iacono T (2002). Health issues for adults with developmental disability. Res Dev Disabil.

[6] Iacono T, Sutherland G (2006). Health screening and developmental disability. J Policy Pract Intellect Disabil.

[7] Cooper S-A, McConnachie A, Allan LM, Melville C, Smiley E, Morrison J (2011). Neighbourhood deprivation, health inequalities and service access by adults with intellectual disabilities: a cross-sectional study. J Intellect Disabil Res.

[8] Glover G, Evision F: *Hospital admissions that should not happen: Admissions for ambulatory care sensitive conditions for people with learning disabilities in England.*2013. http://www.improvinghealthandlives.org.uk/news.php?nid=2228.

[9] Balogh R, Ouellette-Kuntz H, Brownell M, Colantonio A (2013). Factors associated with hospitalisations for ambulatory care-sensitive conditions among persons with an intellectual disability - a publicly insured population perspective. J Intellect Disabil Res.

[10] Balogh R, Hunter D, Ouellette-Kuntz H (2005). Hospital utilization among persons with an intellectual disability, Ontario, Canada, 1995–2001. J Appl Res Intellect Disabil.

[11] Commonwealth of Australia (2011). National Disability Strategy 2010–2020.

[12] PwC: *Use of Emergency and Inpatient Hospital Services by ADHC Clients - Final Report*, Aging Disability and Home Care, Department of Families and Community Services NSW. : ᅟ; 2012.

[13] Backer C, Chapman M, Mitchell D (2009). Access to secondary healthcare for people with intellectual disabilities: a review of the literature. J Appl Res Intellect Disabil.

[14] Iacono T, Davis R (2003). The experiences of people with developmental disability in emergency departments and hospital wards. Res Dev Disabil.

[15] Mencap: *Death by Indifference: 74 Deaths and Counting, a Progress Report 5 Years on.*2012.

[16] Mencap: *Death by Indifference: Following up the Treat Me Right! Report.*2007.

[17] Department of Health (2009). Valuing People now: A new Three-Year Strategy for People With Learning Disabilities.

[18] Michael J, Richardson A (2008). Healthcare for all: Report of the independent inquiry into access to healthcare for people with learning disabilities. Tizard Learn Disabil Rev.

[19] Heslop P, Blair P, Houghton M, Marriott A, Ross L: **The confidential inquiry into premature deaths of people with intellectual disabilities in the UK: a population-based study.** *Lancet* 2013, http://dx.doi.org/10.1016/S0140-6736(13)62026-7. 10.1016/S0140-6736(13)62026-7 24332307

[20] Webber R, Bowers B, Bigby C (2010). Hospital experiences of older people with intellectual disability: responses of group home staff and family members. J Intellect Dev Disabil.

[21] Ailey SH, Hart R (2010). Hospital program for working with adult clients with intellectual and developmental disabilities. Intellect Dev Disabil.

[22] Brown M, MacArthur J, McKechanie A, Mack S, Hayes M, Fletcher J (2012). Learning disability liaison nursing services in south-east Scotland: a mixed-methods impact and outcome study. J Intellect Disabil Res.

[23] Lunsky Y, Tint A, Robinson S, Khodaverdian A, Jaskulski C (2011). Emergency psychiatric service Use by individuals with intellectual disabilities living with family. J Ment Health Res Intellect Disabil.

[24] Sowney M, Barr O (2007). The challenges for nurses communicating with and gaining valid consent from adults with intellectual disabilities within the accident and emergency care service. J Clin Nurs.

[25] NDS: **The experiences of people with disability in NSW public hospitals.** In *NDS Submission: NSW State Plan 2013–2023.*NDS; 2013.

[26] Australian Commission on Safety and Quality in Health Care: *Clinical Care Standards.*2013. http://www.safetyandquality.gov.au/our-work/clinical-care-standards.

[27] NSW Department of Health (2008). People with a Disability: Responding to Needs During Hospitalisation.

[28] Fitzsimmons J, Barr O (1997). A review of the reported attitudes of health and social care professionals towards people with learning disabilities: implications for education and further research. J Intellect Disabil.

[29] Phillips L (2012). Improving care for people with learning disabilities in hospital. [Review]. Nurs Standard February.

[30] Bradbury-Jones C, Rattray J, Jones M, MacGillvray S (2013). Promoting the health, safety and welfare of adults with learning disabilities in actue care settings: a structured literature review. J Clin Nurs.

[31] Downs S, Black N (1998). The feasibility of creating a checklist for the assessment of the methodological quality both of randomised and non-randomised studies of health care interventions. J Epidemiolical Community Health.

[32] Letts L, Wilkins S, Law M, Stewart D, Bosch J, Westmorland M: *Guidelines for Critical Review Form: Qualitative Tudies (Version 2.0).*2007. http://www.srs-mcmaster.ca/Portals/20/pdf/ebp/qualguidelines_version2.0.pdf.

[33] Castles A, Baley C, Gates B, Sooben R: **Experiences of the implementation of a learning disabilty nursing liaison service within an acute hospital setting: A service evaluation.** *Br J Learn Disabil* 2013,

[34] Cumella S, Martin D (2004). Secondary healthcare and learning disability: results of consensus development conferences. J Learn Disabil.

[35] Hart S (1998). Learning-disabled people's experience of general hospitals. Br J Nurs.

[36] Dinsmore A, Higgins L (2011). Study of patients' experiences of treatment by hospital staff. Learn Disabil Pract.

[37] Fox D, Wilson D (1999). Parents' experiences of general hospital admission for adults with learning disabilities. J Clin Nurs.

[38] Gibbs SM, Brown MJ, Muir WJ (2008). The experiences of adults with intellectual disabilities and their carers in general hospitals: a focus group study. J Intellect Disabil Res.

[39] Browne T (1999). A small-scale exploratory study of the needs of learning disabled patients presenting for an x-ray examination. Radiography.

[40] Lunsky Y, Gracey C (2009). The reported experience of four women with intellectual disabilities receiving emergency psychiatric services in Canada. J Intellect Disabil.

[41] Ali A, Scior K, Ratti V, Strydom A, King M, Hassiotis A (2013). Discrimination and other barriers to accessign health care: Perspectives of patients with mild and moderate intellectual disability and their carers. PLoS One.

[42] Winkler D, Sloan S, Callaway L (2007). Younger people in residential aged care: Support needs, preferences and future directions.

[43] Australian Commission on Safety and Quality in Health Care (2012). Annual Report.

[44] Hannon L (2003). Secondary health care for people with learning disabilities. Nurs Stand.

[45] Fyffe C, Bigby C, Bigby C (2012). Specific disability services and the mainstream - What do we mean?. Seventh Annual Roundtable on Disability Policy.

