# Supplementary material for: A systematic review of hospital experiences of people with intellectual disability
Source: BMC Health Serv Res. 2014 Oct 25;14:505. doi: 10.1186/s12913-014-0505-5 (PMC4210514; doi:10.1186/s12913-014-0505-5)
Supplement: Additional file 1: — Summary of studies meeting inclusion criteria. [file 12913_2014_505_MOESM1_ESM.docx]

*Additional File 1.*

*Summary of studies meeting inclusion criteria*

| *Study/ Country* | *Participant Details* | *Appraisal Score* | *Points of Hospital Encounter* | *Interactions Investigated* |
| --- | --- | --- | --- | --- |
| *Mixed Methods* | | | | |
| Iacono & Davis (2003)  Australia | Survey: *n*  = 328, 159 male, 167 female, 18-80 years ( mean = 38), 28 completed by person with disability, 295 by a support person. 223 had ID; 238 a physical disability.  Interviews: *n* = 9, 6 adults with developmental disability, 2 mothers, 1 paid carer. | Quantitative = 6  Qualitative = 3 | ED or wards | Quantitative: not specified  Qualitative: nurses, doctors, including specialists, radiographer |
| *Qualitative* | | | | |
| Hart (1998)  England | 13 people with ID;  5 female; 8 male, 28-49 years (mean = 40.7); 4 mild, 9 moderate ID. | 5 | Inpatients (*n* = 8), outpatient (*n* = 2), day surgery (*n*  = 3) | Not stated, but data refers mostly to nurses, with some relating to doctors |
| Fox & Wilson (1999)  England | 10 parents/ parent couples with adult children 20-49 years (mean 30.4); severe ID, some with physical disabilities | 2 | Orthopaedic wards, ENT, general surgical ward. | Nurses only |
| Browne (1999)  England | 5 people with ID; 6 paid carers | 4 | X-Ray | Largely radiographers, but also some reference to interactions with reception staff. |
| Cumella & Martin (2004)  England | *n* = 80: people with ID, their supporters, family and professional carers, senior managers and professionals in hospital services, commissioners, members of community learning disability teams. | 5 | Not specified | Not specified |
| Hannon (2003)  England | 4 people with ID, mild-severe, 5 family /carers, 5 hospital staff (*n*=6), 5 Learning Disability nurses | 4 | Preadmission | Not stated |
| Sowney & Barr (2006)^a^  Northern Ireland | 27 nurses from 5 hospitals | 6 | A&E | Nurses with patients with ID and carers – types of carers not specified. |
| Sowney & Barr  (2007) ^a^  Northern Ireland | 27 nurses from 5 hospitals | 6 | A&E | Nurses with carers |
| Gibbs et al. (2008)  Wales | 11 people with ID (*n* = 11): 6 female, 5 male, 18 - 62 years; mild-moderate ID, 9 parents, 5 paid carers | 6 | Not identified, but evident from the data that included outpatient clinics, A&E, wards, including surgical. | Not identified, but data focused on interactions with nurses and doctors. |
| Lunsky & Gracey (2009)  Canada | 4 women ID, aged 20s – 40s. | 8 | ED | Mostly nursing, but one reference to interaction with a doctor, one to a paramedic and one with a security guard. |
| Weiss et al. (2009)  Canada | 4 family members, 13 paid carers | 7 | ED for psychiatric crisis | Examples focus on nurse, ED physician and psychiatrist interactions. |
| Webber et al. (2010)  Australia | 17 people with ID, 49-81 years; 17 family members; 16 house supervisors; 11 accommodation programme managers; 11 aged care | 9 | Not stated, but refers to hospitalisation, suggesting had been admitted. | None targeted, but data reflects interactions with a range of hospital staff. |
| Dinsmore (2012)  England | 12 people with ID and their carers | 3 | A&E and various wards | Not specified. |
| Brown et al. (2012)  Scotland | 5 people with ID (*n* = 5), 16 carers. 39 primary care professionals, 19 general hospital professionals, 6  LDLN | 5 | Referral to LDLN | Role of LDLN in relation to patients, carers & hospital staff |
| Ali et al. (2013)  England | 29 people with ID, 23-57 years, 7 males, 22 females, 9 White British or White Other, 3 Asian Pakistani, 2 Asian Indian, 10 moderate/ 4 mild ID; 3 with Down syndrome, 1 cerebral palsy, 2 austism specture disorders; 1 paid carer, 3 partners, 11 mothers, 28-72 years. | 7 | Not specified | Not specified but results focused on GPs and nurses. |
| Castles et al. (2013)  England | 7 people with ID, 3 males, 4 females, 18-66 years (mean = 41); 5 family carers, 5 paid carers, 5 community LD nurses, 1 community LD support worker, 1 care manager; 5 nurses, 4 senior nurses, 1 support worker, 1 physiotherapist, 1 discharge planner. | 4 | Not specified | Not specified |

a = the same study with different themes discussed; ID = intellectual disability; NHS = National Health Service; A&E = accident & emergency; ENT = Ears, Nose & Throat
