# Supplementary material for: A systematic review of hospital experiences of people with intellectual disability
Source: BMC Health Serv Res. 2014 Oct 25;14:505. doi: 10.1186/s12913-014-0505-5 (PMC4210514; doi:10.1186/s12913-014-0505-5)
Supplement: Additional file 2: — Appraisal Checklists. [file 12913_2014_505_MOESM2_ESM.docx]

*Additional File 2*

*Appraisal Checklists*

*Table A: Quantitative*

| ***Reporting*** |  |
| --- | --- |
| 1. Is the hypothesis/aim/objective of the study clearly described? | 1 Yes  0 No  0 Unable to determine |
| 1. Are the main outcomes to be measured clearly described in the Introduction or Methods section?   *If the main outcomes are first mentioned in the Results section, the question should be answered no.* | 1 Yes  0 No  0 Unable to determine |
| 1. Are the characteristics of the patients included in the study clearly described?   *In cohort studies and trials, inclusion and/or exclusion criteria should be given. In case-control studies, a case definition and the source for controls should be given.* | 1 Yes  0 No  0 Unable to determine |
| 6. Are the main findings of the study clearly described?  *Simple outcome data (including denominators and*  *numerators) should be reported for all major findings so*  *that the reader can check the major analyses and*  *conclusions. (This question does not cover statistical tests*  *which are considered below).* | 1 Yes  0 No  0 Unable to determine |
| 10. Have actual probability values been reported (e.g. 0.035  rather than, 0.05) for the main outcomes except where  the probability value is less than 0.001? | 1 Yes  0 No  0 Unable to determine |
| ***External validity*** |  |
| 1. Were the subjects asked to participate in the study representative of the entire population form which they were recruited?   *The study must identify the source population for patients and describe how the patients were selected. Patients would be representative if the comprised the entire source populations, an unselected sample of consecutive patients, or a random sample. Random sampling is only feasible where a list of all members of the relevant population exists. Where a study does not report the proportion of the source population from which the patients are derived, the question should be answered as unable to determine.* | 1 Yes  0 No  0 Unable to determine |
| 1. Were those subjects who were prepared t participate representative of the entire population from which they were recruited?   *The proportion of those asked who agreed should be stated. Validation that the sample was representative would include demonstrating that the distribution of the main confounding factors was the same in the study sample and the source population.* | 1 Yes  0 No  0 Unable to determine |
| ***Internal validity - bias*** |  |
| 18. Were the statistical tests used to assess the main  outcomes appropriate?  *The statistical techniques used must be appropriate to the*  *data. For example non parametric methods should be*  *used for small sample sizes. Where little statistical*  *analyses has been undertaken but where there is no*  *evidence of bias, the question should be answered yes. If*  *the distribution of the data (normal or not) is not*  *described it must be assumed that the estimates used*  *were appropriate and the question should be answered*  *‘yes'.* | 1 Yes  0 No  0 Unable to determine |
| 20. Were the main outcome measures used accurate (valid  and reliable)?  *For studies where outcome measures are clearly*  *described, the question should be answered yes. For*  *studies which refer to other work or that demonstrate the*  *outcome measures are accurate, the question should be*  *answered yes.* | 1 Yes  0 No  0 Unable to determine |
| ***Power*** |  |
| 27. Did the study have sufficient power to detect a clinically  important effect where the probability value for a  difference being due to chance is less than 5%.  *Sample sizes have been calculated to detect a difference*  *of x% and y%.* | 1 Yes  0 No  0 Unable to determine |
| TOTAL SCORE | /9 or 10 |

Note: Adapted from [Downs and Black (1998)](#_ENREF_1)

*Table B: Qualitative*

| ***Reporting*** | *Score* |
| --- | --- |
| *Introduction*   1. Is the research aim/ purpose of the study clearly stated? | 1 Yes   1. No   0 Unable to determine |
| 1. Is the design of the study clearly stated (eg. phenomenology, ethnography, grounded theory)? | 1. Yes   0 No  0 Unable to determine |
| *Methods*   1. Is a method of data collection articulated (eg. Participant observation, interviews, focus groups)? | 1. Yes   0 No   1. Unable to determine |
| 1. Is a process of purposeful selection described? | 1. Yes   0 No   1. Unable to determine |
| 1. Were participants sampled until redundancy is data was reached? | 1. Yes   0 No   1. Unable to determine |
| 1. Is there a clear description of data collection (including gaining access to data collection, methods, time spent, amount of data)? | 1. Yes   0 No   1. Unable to determine |
| 1. Were data analyses inductive (findings emerged from the data)? | 1. Yes   0 No  0 Unable to determine |
| 1. Was a decision trail reported so that rules and decisions made in the analyses are clear? | 1. Yes   0 No   1. Unable to determine |
| 1. Was the process of transforming data into themes/ codes adequately described? | 1. Yes   0 No  0 Unable to determine |
| 1. Is there evidence for the 4 components of trustworthiness:   Credibility *(a true picture has been presented)*  Transferability *(enough details provided to determine if findings can be transferred to other situations)*  Dependability *(audit trail is clear so the consistency between data and the findings can be seen)*  Confirmability *(strategies to limit bias in the research can be seen, such as use of reflective journal, member checking)* | 1. Yes, all   1 Evidence of one at  least but not all four  0 No  0 Unable to determine |
| ***TOTAL SCORE*** | */11* |

Note: Adapted from [Downs and Black [1]](#_ENREF_1), Letts et al. [2], PEDro [3]

*References*

1. Downs S, & Black N: **The feasibility of creating a checklist for the assessment of the methodological quality both of randomised and non-randomised studies of health care interventions.** *Journal of Epidemiolical Community Health,* 1988, ***52*:** 377-384. doi: 10.1136/jech.52.6.377
2. Letts L, Wilkins S, Law M, Stewart D, Bosch J, & Westmorland M: **Guidelines for critical review form for qualitative studies: Version 2.** 2002, Retrieved 8^th^ July, 2013 from http://www.canchild.ca/en/canchildresources/resources/qualguide.pdf
3. PEDro. **Physiotherapy evidence database.** Retrieved 8^th^ July, 2013 from http://www.pedro.org.au
